# International women’s day in Nigeria: the paradox of gender visibility and inequality

**DOI:** 10.3389/fsoc.2026.1963880

**Published:** 2026-09-17

**Authors:** Samson Uchenna Eze

**Affiliations:** Music Department, Wesleyan University, Middletown, CT, United States

**Keywords:** corporate co-optation, gender inequality, gender visibility, international women’s day, Nigeria, patriarchal femocracy

## Abstract

Nigeria’s celebration of International Women’s Day (IWD) has evolved from the era of federal government observances in the 1980s to corporate commemorations. Nigeria is the most populous country in Africa, where IWD is celebrated annually amid a huge number of women affected by gender-based discrimination. In decades of expanding women’s awareness, IWD has witnessed a mix of celebrations, commercial fanfare, and protests. It has become a platform for belonging politics, show of achievement, and commercial mobilization. In all of this, the gender gap remains grossly disproportionate. Amid many challenges facing women in Nigeria, IWD is celebrated with images and presence of accomplished women. This study argues that Nigerian government appropriates IWD into state legitimation exercises and uses the day to publicize social welfare programs without addressing the underlying gender inequality. In addition, corporate commercialization of IWD in Nigeria has not addressed structural inequality. Demonstration of achievement for a few women is not tantamount to overall women’s liberation, and Nigeria’s IWD often substitutes the show of individual achievements for collective liberation. Drawing on a focus group discussion with twelve Nigerian women and field semi-structured interviews conducted during IWD events in Nigeria, the study situates its analysis on women’s testimonies. This study concludes that while flamboyant IWD celebrations are good for women in their own terms, Nigerian women must shun patriarchal femocracy and sustain protests given the marginal position of women in the nation.

## Introduction

International Women’s Day, which began in 1911 with women activists in Germany, Denmark, Austria, and Switzerland, gained formal United Nations recognition in 1977, when the General Assembly invited member states to support women’s rights. International Women’s Day celebrates women’s gains and reminds women about persistent structural gender inequalities and strategies for challenging them (LeSavoy and Jordan, 2013). According to the 2026 United Nations call for IWD, women and girls across the world must demand equal rights and the justice needed to enforce, exercise, and enjoy those rights. The UN statement notes that no country has succeeded in closing the legal gap between men and women, as women hold a pinch of the rights afforded to men globally. The UN observes that across the fundamental domains of life, women are at disadvantage positions (United Nations, 2026).

It is important to note that IWD reached Nigeria as an external ritual, already encoded with Global North’s ideology of women’s liberation. Nigeria is a country whose women’s movements have their own indigenous histories. For instance, the Aba Women of 1929 and the Abeokuta Women’s Union (led by Funmilayo Ransome-Kuti in the 1940s and 1950s) made their mark as advocates in southeastern and southwestern Nigeria (Byfield, 2021; Mba, 1982; Van Allen, 1972). Currently, capitalist interests shape women’s feminist action in Africa (Udenigwe et al., 2026). In Nigeria, Africa’s most populous nation and largest economy, IWD evolved from state commemorations into a mix of protests, corporate conventions and speeches, gala dinners, hashtag campaigns, and complimentary commercial initiatives. Within this public sphere, overall women’s economic, educational, and political welfare continues to fall.

The Global Gender Gap Report of 2025 ranked Nigeria 124th out of 148 nations, given that women hold 3.91% of seats in the National Assembly and 8.82% of ministerial positions. Of the 148 countries, Nigeria’s political empowerment of women ranks 143rd. Political representation of Nigerian women declined from 17.6 to 8.8%, while in educational attainment, men’s rates increased to 73.7% while women’s rates slid down to 53.3% (World Economic Forum, 2025). Nigerian women are systematically excluded from the institutional spaces where key decisions are made. In governance, women’s organizations pursue gender equity, but these efforts have not translated into substantive political representation (Effevottu, 2021). In the security structure, women bear the burden of violence and displacement and are absent in the leadership of security agencies (Afolabi, 2019, 2022). According to Punch News femicide report, 197 women and girls lost their lives between January 1 and December 31, 2025 (PunchNews, 2026).

This study does not discountenance Nigeria’s IWD celebration. Women’s jubilation for some progress made is encouraged. However, it offers a critical and objective review of the celebration amid challenges that continue to impact women negatively. This study interrogates the celebration of IWD and asks which institutions shape its meaning. What roles do the Nigerian state, corporate establishment, and faith-based institutions play in the legitimation, recognition, and commodification of International Women’s Day?

### Visibility and the limits of liberation

This study draws from feminist theories that explain the paradox of gender visibility and social inequality. Drawing from the work of Fraser (1995), Hooks (1992), Crenshaw (1989), and McRobbie (2008), this study argues that the flamboyant visibility of women in public, institutional, and cultural domain does not constitute or guarantee attainment of structural gender equality. Visibility, under unequal conditions and material exclusion, reinforces the hierarchies they appear to challenge (Hooks, 1992).

Fraser (1995) has critiqued the quest for recognition without redistribution of resources. Fraser (1995, 70) calls this “the recognition-redistribution dilemma,” arguing that the pursuit of recognition or visibility can, under the conditions of capitalism, displace demands for economic justice. Fraser’s model explains contemporary feminist, progressive movements that have oriented themselves toward recognition at the expense of redistribution. As a result, institutional representation of women and greater media visibility may suggest progress without dismantling the unequal structural conditions that sustain women’s subordination. In Nigerian IWD, the spectacle of ministerial briefings and bank conferences represents this substitution of recognition for redistribution. The critique of representation extends to Hooks’s (1992) analysis of corporeal commodification of Black women. Hooks argues that women’s representation and visibility in the media do not inherently constitute women’s liberation. Arguing from the perspective of Black women’s visibility (sometimes sexualized) in Western media, hooks posits that sexist and racial tropes of such representations undermine women’s collective and individual liberation. Beyond sexualization, hooks critiques commodified visibility, asking whose images are projected and for what purposes. The media representation of a few Nigerian women on IWD as “liberated” figures is more of a commodified spectacle that silences the patriarchal, economic, and sociocultural obstacles.

Crenshaw (1989, 1991) discusses the selectivity and internal hierarchies of feminist and gender representation, arguing that visibility of gender in cultural or political discourse favors the most privileged. Crenshaw (1991) enjoins us to rethink whose visibility and progress are amplified and who continues to be suppressed. McRobbie (2008) has cautioned that feminist gains and public visibility of some women are acknowledged and deployed as grounds for declaring political feminism obsolete and unnecessary. Under this postfeminist framework, the visibility of successful women in media, professional life, and popular culture is projected as evidence that equality has already been achieved. In Nigeria’s IWD, images of many prominent women on bank billboards and conference halls demonstrate the commodification of women’s achievement or institutional co-optation. Every year, like the Zenith Bank’s (2026) IWD event, the images of Ngozi Okonjo-Iweala and Chimamanda Ngozi Adichie are printed boldly in IWD conference halls in Lagos. In this way, feminist demands are neutralized by neoliberal capitalist, and patriarchal structures that foreclose the conditions for collective political action.

In sum, Nancy Fraser warns us that recognition without redistribution breeds structural inequality. Bell hooks posits that visibility controlled by dominant media structures commodifies women with no liberation. Crenshaw’s model shows that gender visibility is always selective, centers the privileged women and renders others doubly invisible, while McRobbie warns that visibility is mobilized to displace and depoliticize feminist critique. These concepts guide this study in analyzing Nigeria’s IWD gender visibility as symbolic inclusion within structural exclusion.

IWD in Nigeria has largely maintained a stance of femocracy amid the underlying gender gap and inequality across the board. Mama (1995, 41) characterizes femocracy in Nigerian women’s struggle as “an anti-democratic female power structure, which claims to advance the welfare of ordinary women but is unable to do so because it is dominated by a small clique of women whose authority derives from their being married to powerful men.” This patriarchal femocracy, Mama (1995) argues, “exploits the commitments of the international movement for greater gender equality while actually only advancing the interests of a small female elite, and in the long-term undermining women’s interests by upholding the patriarchal status quo.” Mama concludes that “femocracy is a feminine autocracy running in parallel to the patriarchal oligarchy upon which it relies for its authority” (Mama, 1995, 41). Annually, IWD spectacle in Nigeria (staged by the state, corporate establishment, and patriarchal femocracy) neutralizes collective feminist politics and constitutes ceremonial inclusion amid structural exclusion.

## Methodology

This qualitative study employed the historical descriptive design. Views of Nigerian women during the 2022 IWD were analyzed alongside news reports and corporate publications on the 2025 and 2026 IWD celebrations. The years examined were determined by the availability of data on commemorative activity. The 2022 IWD witnessed street protests, while 2023 and 2024 IWD events favored restful state, faith-based, and corporate commemorations. The field interviews were conducted with 45 respondents during the 2022 International Women’s Day events in Abuja and Lagos, Nigeria. Data were also gathered from an Abuja focus group comprising twelve Nigerian women. Participants were sampled purposively to cover organizers and other participants or protesters. The focus group was selected to reflect a diversity of occupational backgrounds and age (ranging from 21 to 61 years). The focus group and field interview respondents included civil servants, market traders, NGO workers, domestic workers, journalists, secondary school teachers, students, and unemployed university graduates. Participants were members and non-members of women’s organizations. Each participant understood the purpose of the research, and each provided oral informed consent. Anonymity of participants was also maintained in discussion. Focus group sessions were audio-recorded with participants’ permission and subsequently analyzed. The field interviews were designed to elicit spontaneous, unmediated responses to the day’s events and to complement the focus group data. Interviews were conducted in English and, in a few cases, Nigerian Pidgin English. All responses were analyzed using descriptive approach and content analysis.

### Nigerian government and the IWD

Nigeria’s official IWD event was popular during the Babangida regime. Between 1985 and 1999, IWD in Nigeria was more of a federal government’s affair. First Ladies, especially Maryam Babangida, whose Better Life for Rural Women program began in 1987, and Maryam Abacha, whose Family Support program followed in 1994, used the day to project a maternal vision of women’s development through rural women empowerment initiatives (Mama, 1995). Women’s maternal role as peacemakers was summoned in preparation for the 1992 International Women’s Day celebration. At the time, Nigeria’s First Lady, Maryam Babangida, worked with Nigerian female music icon, Onyeka Onwenu, to promote the “Peace Song.” The song depicts Nigerian women as peace makers:

We’re the women of Nigeria

And peace is what we bring

Let it reign, let it reign everywhere

We’re the children of Nigeria

And peace is what we need

So that all can live as one (Peace Song, Onyeka Onwenu, 1992).

The “Peace Song” portrayed Nigerian women as selfless mediators as Onwenu invoked the image of motherhood given the cultural significance of mothers in Nigerian/African communities. Through the song, the First Lady promoted women as mothers who initiate peace beginning from the family unit to the national stage. Although the lyrics emphasize the maternal peace as key to good governance, no woman has been elected as a state governor in Nigeria since 1999, and this reflects the pervasive gender disparity.

IWD events organized by the government promote the ruling administration’s program and suppress issues that inconvenience the political coalition such as parliamentary gender equality. The Nigerian state has, across successive administrations, used International Women’s Day as a stage for showcasing the gender-related programs. This behavior cuts across Better Life for Rural Women program of the late 1980s, Family Support program of the mid-1990s, National Gender Policy reform efforts of the 2000s, all of which were tied to welfare schemes of the Federal Ministry of Women Affairs and National Center for Women Development.

The women’s welfare programs, criticized by feminist scholars as elite philanthropy that did little to unsettle patriarchal structures, established IWD as a ceremonial stage for the wives of the head of state (Mama, 1995). At the time, annual IWD ceremonies in Lagos and Abuja during the 1990s were choreographed, state-organized women’s wings, with banners reading “Better Life for Rural Women,” radio broadcasts and televised speeches from the First Lady. The military regimes used the day to demonstrate that the state had not abandoned women, even as structural adjustment policies under the International Monetary Fund eroded the urban middle class and pushed more women into poverty. This contradiction (celebratory speeches amid deteriorating women’s health, economic, and political advancement) would become a structural feature of the Nigerian IWD.

Since the return of civilian rule in 1999, Nigerian First Ladies have sustained the tradition. Every March 8, Nigeria’s Ministry of Women Affairs holds the ministerial press briefing. The March 7, 2026 edition, organized by Minister Imaan Sulaiman-Ibrahim, was no different. According to the United Nations, the 2026 IWD activities were held under the theme “Right. Justice. Action. For all Women and Girls,” a rhetoric that echoes the urgency of social transformation. There was no action from Nigerian women groups in form of protests. The women’s ministry outlined all its work along with the “Renewed Hope” campaign mantra of President Bola Tinubu. Many of the focus group respondents opined that such a politicized IWD subordinates women’s action to political loyalty. Accordingly, a lady who works with the women’s ministry stated, “every year our programs put food on the table for some women, we train women and support widows and rural women. The progress is worth celebrating, and I am proud to be part of it”.

IWD in Nigeria expanded into NGO or civil society organizations. Groups such as the International Federation of Women Lawyers (FIDA Nigeria), the Women’s Rights Advancement and Protection Alternative (WRAPA), the Women’s Consortium of Nigeria, and the Women in Management, Business and Public Service (WIMBIZ) organize talks on March 8 and hold health, and legal-aid sessions. Regrettably, these civil society organizations work with every incumbent patriarchal government. In her discussion of “wifeism and activism” in Nigerian women’s movements, Abdullah (1995, 224) posits that Nigerian government sees these organizations as wives in the traditional sense that they must follow the dictates of the government. Abdullah argues that “a true feminist agenda challenges the state and if the Nigerian women’s movement seeks emancipation, it must divorce itself from the grip of the state”.

It is worth comparing the Nigerian record with other countries, since IWD is celebrated everywhere in Africa. Rwanda, a country whose women hold over 60 percent of parliamentary seats following constitutional reforms, has achieved a structural transformation that Nigeria has not (Abouzeid, 2019). South Africa enshrined gender equality in its post-apartheid constitution and has produced female heads of major parastatals, although gender-based violence persists. The annual IWD coverage in Nigerian newspapers and on Nigerian Twitter (now X), is voluminous, but the parliamentary and judicial breakthroughs are not commensurate.

### The commercialization of Nigerian IWD

Corporate co-optation of IWD has been a topic in global feminist discourse. LeSavoy and Jordan (2013) argue that International Women’s Day in Russia and the United States has been hijacked by corporate marketing interests. They argue that corporate interests converted a historically radical platform for women’s rights into a means for commodity consumption and brand promotion. Though the commercialization of IWD works differently across national contexts, it produces the same results, which is the neutralization of feminist political demands (LeSavoy and Jordan, 2013). The culture of “femvertising” in the West, where companies co-opt IWD and Valentine’s Day to woo women to their products, has become a global patriarchal depoliticization mechanism. Cox (2019) questions the volume of femvertising emails that buzz women’s phones across the world in the build-up to March 8. Cox laments that advertisement of products unrelated to IWD can be “unflattering and insulting.” Bluntly, Cox believes “companies that co-opt IWD should be ashamed.” Diana Kussainova writes that for brands, “International Women’s Day marketing is a chance to drive sales and make more profit. But at the same time, businesses can channel their support towards a good cause and highlight the achievements, challenges, and stories of their customers” (Kussainova, 2026, 1).

Currently, corporate sponsors, and brand activations have turned Nigeria’s IWD into an annual marketing season comparable to Valentine’s Day. According to Bellanaija (2026, 2) “IWD is a big deal in Nigeria. To commemorate the day, corporate organizations often host events and gift female employees, brand and marketing managers, partners, and generally women in society.” For the 2025 IWD, Promasidor Nigeria (PNG), the producers of Cowbell milk stated that during their event, “attendees were dressed in beautifully branded PNG Women T-shirts” (Promasidor, 2025, 5).

The commercial banks and the churches have also redefined IWD in Nigeria. Many Nigerian commercial banks celebrate and market their initiatives on or around March 8. Zenith Bank’s event is its annual International Women’s Day seminar. The bank’s 2026 IWD celebration in Lagos had the governor of Lagos’s wife amid other dignitaries. The governor’s wife’s speech to the elite women in the hall did no less than advance the governor’s promises of addressing women’s challenges. This is the ritual of femocracy in Nigeria’s IWD celebration. One of the discussants, a trader, remarked that the celebrations are meant for the rich women in heels and expensive gowns, while for traders in the market, every day is a struggle. Zenith bank had its own theme, “Take It, You Own It” along with the global “Give to Gain” campaign (Zenith Bank, 2026). The Chief Executive Officer, Adaora Umeoji, structured the bank’s 2026 IWD activity as part of an effort to empower women (Financial Nigeria, 2026).

During the Zenith Bank’s IWD conference, Nigerian Afrobeats artist, tomboy Teni, eulogized the CEO Adaora Umeoji, describing her as a wealthy woman. The CEO quickly responded by telling the artist, “I save money; I keep your money safe.” This short narrative on stage describes the encoding of profit-making gestures in Nigerian banks’ IWD celebrations. The theme, “Take it, You Own It,” is encoded with credit propositions for women. One of the participants stated, “sitting in that hall and watching female CEOs share their journey moved me deeply. My daughter watched the livestream and saw what could be possible.” In April 2026 the bank, in partnership with the Ford Foundation, also launched an inaugural “Woman of Power” award that honored nineteen Nigerian women (News Online Nigeria, 2026).

Access Bank’s IWD structure is built around “The W Initiative,” launched in July 2014. It is reported that the health program has impacted women through free screenings for breast and cervical cancer, eye, and blood pressure (Moses-Ashike, 2022). The Access Bank’s W initiative for Nigerian women, also tagged Maternal Health Service Support, while aiming to solve a public health problem, places women on loans for child delivery. The loan is granted after checking the applicant’s credit history. So, a woman who has a poor credit history is unlikely to be granted the maternal health support loan. The initiative risks deepening inequality because creditworthy women are likely to access the loan. These initiatives are real in the limited sense that they do disburse some credit, host some events, and promote prominent women. But they are also marketing in a strict sense.

In a country where maternal healthcare costs are expensive, the scheme eases the financial burden associated with accessing fertility treatments, a service the average Nigerian woman cannot afford out-of-pocket. The initiative, however well-intentioned, transfers the burden of the Nigerian government onto women and their families. Childbirth and maternal care are subtly marketed on IWD celebrations through screen displays and speeches of bank executives. This way, the bank profits from a systemic failure in state healthcare. One of the discussants, a secondary school teacher, observed that though banks celebrate women on IWD, some banks seldom grant her a loan without proof of a guarantor (preferably an employed male guarantor or husband).

Access Bank also runs a promotional campaign called “The Power of 100,” which recognizes one hundred outstanding women across Nigeria and Africa. The 2025 IWD edition culminated in an event that honored Ngozi Okonjo-Iweala, Director-General of the World Trade Organization (Imoh, 2025). Amid celebrations, credit products are encoded to appeal to the creditworthy demographic of urban professional women.

On March 8, Nigerian women are gathered into stadiums and hotel ballrooms, photographed beside government officials and corporate executives, and told that they belong. They are reminded of Funmilayo Ransome-Kuti, Margaret Ekpo, Hajiya Gambo Sawaba and other influential women in history. Each year a small circuit of high-profile women grace the IWD panel with expensive dresses, a keynote address, and their presence lends the event the moral credence of female accomplishment.

As Nigerian elite women (governor’s wives, actors, bank executives) gather in stadium and banquet halls for the annual celebration of IWD, class is pronounced in sartorial features, heavy security presence, and absence of the average market women. This leads to the question, who is Nigeria’s IWD celebration for, who is in the banquet hall in Lagos and Abuja and who is not? Not in the room are the women whose statistical condition is the reason IWD exists. The discussants mentioned the farmers whose farms were overrun by cattle in Benue, the female students in Borno displaced by Boko Haram violence, and the survivors of sexual violence in Kano. As elite women place orders for designer wears to “oppress” other women on IWD events, how many women can place new orders for the day’s appearance? One of the discussants, a government official, argued that visibility of successful women on International Women’s Day sends a signal to Nigerian government and institutions that women belong in leadership. She maintained that women cannot demand social change from a position of total invisibility, and that the celebration of accomplished women unsettles the sociocultural norms that undermine young women’s career aspirations.

### The faith-based institutions on IWD

The Nigerian Christian churches play a key role in promoting the annual IWD celebrations. The Redeemed Christian Church of God, Living Faith Church (Winners’ Chapel), Daystar Christian Centre, House on the Rock, and Dunamis International Gospel Centre, among many others, hold special activities and sermons in the days surrounding March 8. These churches draw congregations in the thousands, and deploy the language of empowerment, destiny, and divine purpose in the same manner commercial banks use the language of financial inclusion.

The theological framing of the church’s IWD positions Nigerian women as biblical models of industriousness, domestic virtue, and entrepreneurial energy who are devoted wives, capable traders, and women of faith. The Nigerian woman’s figure is honored loudly and sincerely on IWD. This “ideal woman” is celebrated as a figure whose empowerment is compatible with the patriarchal structure of marriage and church authority. The church and the postcolonial African states celebrate the woman who maximizes her potential within the patriarchal order and do not celebrate or accommodate the woman who challenges the order (Tamale, 2020). Whereas banks offer credit to some women, churches offer prayers, consecration, and prophetic declarations. In some churches, the success of each year’s IWD celebration is measured by funds raised by women groups in the church.

Within these churches, senior pastors’ wives serve as the face of women’s department, representing, in a theological aura, the First Lady figure that characterizes Nigerian state IWD since Babangida’s military era. The structural parallel in both cases is the sustenance of a powerful patriarchal institution that portrays the wife of its leader as the symbol of women’s advancement, while the leadership remains male-dominated. As Mama (1995, 42) has observed, the Nigerian state has sustained an all-male colonial structure of governance to control the population and the women. The megachurches’ IWD depoliticizes women’s conditions through theological sensation.

The church’s IWD, like the bank’s, performs one version of belonging. The woman who attends a church IWD conference is told she is fearfully and wonderfully made, that her gifts are divinely ordained, and that her season of breakthrough is at hand. Such words of affirmation provide no reform agenda or a legislative demand. It defers social transformation and relegates the solution to women’s problems to the spiritual. During the field discussion, a female pastor in Lagos stated, “at our IWD service, testimonies flow, and women go home healed, restored, and reminded of their worth. The church offers little physical gifts, but the spiritual is priceless.”

The commercial bank and the church speak the language of finance and faith, respectively. These two sectors promote women’s empowerment as an individual achievement to be unlocked through the right credit, program, prayer, and virtue. As Cornwall and Brock (2005) argue, “empowerment” has become such a buzzword in development discourse that often displaces examination of structural inequality in favor of individual success promotion. In Nigeria’s IWD context, the bank seminar and the church conferences accomplish this displacement.

The commercialization of International Women’s Day in Nigeria extends beyond the banking sector and the church into the real estate industry, where property companies have annexed March 8 as a marketing opportunity. In some Nigerian states, real estate ladies visit secondary schools and distribute sanitary pads to adolescent girls while wearing corporate t-shirts bearing the logos and property listings of their companies. The gesture is presented as social responsibility and women’s empowerment, but it is a calculated brand visibility exercise targeting young women (and their parents).

### Protest and celebration

While government officials in Abuja have long structured International Women’s Day as a day for compliments, Nigerian women on March 8, 2022, chose a different kind of commemoration. The March 8, 2022, protest was a direct indictment of the state’s failure to advance women’s inclusion in governance. The protest emerged in response to the legislature’s rejection of gender equality bills (Eniola, 2018). The gender bills sought to provide special seats for women at the National Assembly, implement 35% affirmative action for women in political party administration, grant citizenship to foreign-born husbands of Nigerian women, and allow married women to be elected in their husbands’ states of origin (Orisadare, 2019).

The protest had civil society organizations such as Action Aid Nigeria, the Nigerian Association of Women Journalists (NAWOJ), and Christian Women in Nigerian Politics. Many other women’s organizations like the Womanifesto, Women Advocates Research and Documentation Center (WARDC), National Coalition on Affirmative Action (NCAA), Gender Mobile, and Gender and Constitution Reform Network (GCRN), were all involved. Women sang as they marched to Nigeria’s National Assembly complex in Abuja. The DJ played songs that tell stories of women’s subjugation. Of note is Simi’s song “Woman” (2021), which questions the enduring subjugation of Nigerian women:

#### Pidgin lyrics

Woman don suffer o/Lowo everybody…/If woman never marry oh, then she no be woman/If woman never carry omo, she no be woman/If she no get man for side oh/Dem go dey push woman aside oh/And eventually oh/Dem go suffer suffer woman…/ Woman, woman, woman, nobody be like woman…/Na so submission oh/Shey we get assignment?/But you no pay attention oh/To the intimidation she dey face

#### English translation

Woman has suffered/In the hands of everybody…/An unmarried woman is not respected as a woman/If a woman has not given birth, she is not a woman/If she does not have a man by her side/Society will disregard her/And eventually/They will continue to disregard her…/Woman, woman, woman—nobody is like a woman/Everyone expects us to always be submissive/And this is our major assignment?/However, nobody pays attention/No one pays attention to the discrimination and intimidation she faces (Eze, 2026, 5-6).

Simi’s “Woman” song became a song of choice for the protest because it narrates the entrenched gender-based discrimination in Nigeria. It decries sociocultural apathy to women’s struggles with discrimination and violence. One of the focus group discussants argued that the Nigeria’s IWD is an event, in which women’s organizations enact a feminist commitment for a single day before returning to their male-dominated work domains.

The United Nations theme for 2022 IWD (Gender Equality today for a Sustainable Tomorrow) aligned with women’s protest for Nigeria’s legislative position. Some Nigerian male politicians questioned the rejection of the bill. A statement credited to the former governor of Rivers State, Nyesom Wike, reads, “It is only when Nigeria recognizes you that you can dance to celebrate International Women’s Day” (NewsDigestNG, 2022). While Wike’s views at the time may be read as the language of oppositional party politics, it reflects the views of many focus group participants in this study.

One of the discussants expressed her frustration, noting that Nigerian women have been celebrating International Women’s Day since the Babangida years, and yet the National Assembly rejected the gender equality bills in 2022. She asked when women would stop celebrating and start legislating. Another discussant, a younger university student, recalled the urgency of that season and affirmed that protesters felt that if they did not speak up before the 2023 elections, post-election protest would yield no results.

The pre-election season necessitated the urgency for protests. With the 2023 general elections around the corner, 2022 was a realistic opportunity for constitutional reform before new officials would take over. Though women constitute a large chunk of Nigeria’s voting population, less than 5% of over a thousand women who contested the 2023 general elections were elected (BlueprintNG, 2023). The poor performance of women in public service is rooted in Islamic, colonial, and Christian views that women are created as subordinates to men, a condition which the military governments used to undermine women’s liberation (Dennis, 1987).

Amid protest in the morning hours of March 8, 2022, some Nigerian elite women received awards in the evening in Lagos. At the gala award event organized by UN Women in conjunction with UNDP, British High Commission, European Union, and the embassies of France, Germany, and the United States, Kate Henshaw, a Nollywood actor, seized the opportunity to protest the National Assembly’s rejection of the gender bills.

Since the 2022 protest at the National Assembly complex, agitation for the consideration of the gender bill appears to have gone under the table. However, in March 2025, the suspension of Senator Natasha Akpoti-Uduaghan on 6 March, just two days before IWD, sparked protests in Abuja. Natasha, one of only four women in Nigeria’s 109-member Senate, accused Senate President Godswill Akpabio of sexual harassment. The Senate suspended her for six months without investigating the allegation. Protesters took to the streets with placards reading, “We are all Natasha.” Women protesters and counter-protesters all turned the issue into the rave of the moment (CBSNews, 2025; VanguardNews, 2025; Eze, 2026).

In Lagos on 8 March 2025, thousands of women filled the Mobolaji Johnson Stadium, dancing and celebrating with the state government’s support. Although the state governor and his wife gave speeches promising gender equality, Natasha’s case was not mentioned. For the governor, addressing allegations of sexual harassment in the Senate would be an attack on a fellow ruling party member/man, Akpabio. Natasha was in the opposition party. Governors across the states and elite women remained mute, but Natasha took the matter to the United Nations. In New York, Natasha narrated her experience at the Inter-Parliamentary Union meeting of the United Nations’ Women in Parliament. Nigerian IWD events, which failed to protest Natasha’s case in the Senate, were [and still are] controlled by patriarchy.

## Conclusion

The statistics on political representation, financial inclusion, education, femicide, employment, and maternal mortality contradict the elaborate elite celebratory vigor of Nigeria’s IWD. This is not an argument against the International Women’s Day celebration. However, it challenges the government, the influential elite women to rethink progress made so far. A socially conscious IWD would continue to ask why the National Assembly rejected the gender equality bills in 2022. Until then, Nigeria’s IWD will continue to be a day the patriarchal system renews its promises of gender equality. The question is not about the loudness of celebration, but whether Nigerian society is prepared to convert that celebration into social and legislative reform. This is the unfinished work of the Nigerian feminist struggle.

The Nigerian churches and commercial banks’ IWD programs are not sustainable feminist interventions. In the absence of government’s social reform, corporate co-optation cannot solely address structural inequality. Nigerian banks’ IWD initiatives are useful, but they are not, by themselves, instruments of women’s liberation. The banks’ IWD programs promote the bank’s importance and suppress the political demand for public alternatives. Churches use the IWD celebration to raise funds and reassure women that through faith, they can work and pray their way to success. The megachurches hardly mention the marginal position of women in the church or the society at large. For many pastors who remain politically correct, issues of gender parity that unsettle the ruling government are distanced from the sermon on the pulpit.

Given the pervasive gender gap in Nigeria, IWD must continue to be a day for protests. This study proposes an initiative for a “Nigerian Women’s Day,” which can augment IWD. From a historical perspective, “Nigeria Women’s Day” would be celebrated (with protests) on November 23, being the very day Aba women began the 1929 confrontation with colonialists and rejected the taxation of market women (Van Allen, 1972). In 2026, Abia State governor Alex Otti announced the state government’s plans to honor the revolutionary Aba women with monuments.

Given the insecurity challenges in accessing remote villages, the study could not cover the input of women such as farmers whose lands are overrun in Benue state and the displaced women in IDP camps (internally displaced persons) in Borno state. Further research may shift away from the metropolitan IWD space into the rural communities affected by armed conflict in northern Nigeria. The discussion of faith-based institutions in this study favors Nigerian Christian churches. Since Nigeria is a Christian-and-Moslem majority state, further research may explore how women within the Moslem faith celebrate IWD within mosques and other Islamic spaces. Comparative research across Anglophone West African states would establish whether Nigeria’s co-optation of IWD (by the state, corporate, and faith-based institutions) is a shared regional phenomenon.

## Data Availability

The raw data supporting the conclusions of this article will be made available by the authors, without undue reservation.

## References

[ref1] Abdullah H. (1995). “Wifeism and activism: the Nigerian women’s movement,” in The Challenge of Local Feminisms: Women’s Movements in Global Perspective, ed. Basu A. (Boulder: Westview Press), 209–225.

[ref2] Abouzeid R. (2019). How Women are Stepping up to Remake Rwanda. Available online at: https://www.nationalgeographic.com/culture/article/how-women-are-remaking-rwanda-feature (Accessed May 1, 2026).

[ref3] Afolabi C. Y. (2019). The invisibility of women’s organizations in decision making process and governance in Nigeria. Front. Sociol. 3:40. doi: 10.3389/fsoc.2018.00040

[ref4] Afolabi C. Y. (2022). Women and insecurity in Nigeria: the way forward. Front. Sociol. 7:734190. doi: 10.3389/fsoc.2022.734190 PMC9301453 35874447

[ref5] Bellanaija (2026). International Women’s Day 2026: How Meta4’s Lady Flora Represents IWD in Nigeria. Available online at: https://www.bellanaija.com/2026/02/meta4s-lady-flora-represents-iwd/ (Accessed April 5, 2026).

[ref6] BlueprintNG. (2023). Gender Bills: What Women want from Lawan, 9th NASS. Available online at: https://blueprint.ng/gender-bills-what-women-want-from-lawan-9th-nass/ (Accessed January 3, 2025).

[ref7] Byfield J. A. (2021). The Great Upheaval: Women and Nation in Postwar Nigeria. Ohio: Ohio University Press.

[ref8] CBSNews (2025). Nigeria Female Senator Natasha Akpoti-Uduaghan Suspended after Filing Sexual Harassment Claim against Senate President Available online at: https://www.cbsnews.com/news/nigeria-senator-natasha-akpoti-uduaghan-suspended-sexual-harassment-godswill-akpabio/ (Accessed December 5, 2025).

[ref9] Cornwall A. Brock K. (2005). What do buzzwords do for development policy? A critical look at ‘participation,“empowerment’ and ‘poverty reduction.’”. Third World Q. 26, 1043–1060. doi: 10.1080/01436590500235603

[ref10] Cox J. (2019). The Companies who co-opt International Women’s Day should be Ashamed. Available online at: https://www.theguardian.com/commentisfree/2019/mar/08/companies-international-womens-day-equality-feminism (Accessed May 25, 2025).

[ref11] Crenshaw K. (1989). Demarginalizing the intersection of race and sex: a black feminist critique of antidiscrimination doctrine, feminist theory and antiracist politics. Univ. Chic. Legal Forum 1, 139–167. Available online at: http://chicagounbound.uchicago.edu/uclf/vol1989/iss1/8

[ref12] Crenshaw K. (1991). Mapping the margins: intersectionality, identity politics, and violence against women of color. Stan. Law Rev. 43, 1241–1299. doi: 10.2307/1229039

[ref13] Dennis C. (1987). “Women and the state in Nigeria: the case of the Federal Military Government 1984-85,” in Women, State and Ideology: Studies from Africa and Asia, ed. Afshar H. (New York: State University of New York Press), 13–27.

[ref14] Effevottu E. S. (2021). “Women’s political representation and sustainable development in Nigeria, 1999–2019.” Young African Leaders Journal of Development 3:23–29. Available online at: https://digitalcommons.kennesaw.edu/yaljod/vol3/iss1/5

[ref15] Eniola B. O. (2018). Gender parity in parliament: a panacea for the promotion and protection of women’s rights in Nigeria. Front. Sociol. 3:34. doi: 10.3389/fsoc.2018.00034

[ref16] Eze S. U. (2026). “Nobody be like woman”: popular music and the discourses of gender justice in Nigeria. Feminist Media Stud., 1–19. doi: 10.1080/14680777.2026.2620769

[ref17] Financial Nigeria. (2026). Adaora Umeoji and Gender in Nigerian Banking Leadership Available online at: https://www.financialnigeria.com/adaora-umeoji-andgender-in-nigerian-banking-leadership-blog-864.html/ (Accessed April 2, 2026).

[ref18] Fraser N. (1995). From redistribution to recognition? Dilemmas of justice in a post-socialist age. New Left Rev 1, 68–93. doi: 10.64590/4rl

[ref19] Hooks B. (1992). Black Looks: Race and Representation. Boston: South End Press.

[ref20] Imoh R. (2025). IWD: Access Bank Honours Okonjo-Iweala, hail Women, Promises Empowerment. Available online at: https://freedomonline.com.ng/iwd-access-bank-honours-okonjo-iweala-hail-women-promises-empowerment/ (Accessed May 5, 2026).

[ref21] Kussainova D. (2026). 20+ Creative Women’s Day Campaign Ideas for Brands (2026 Edition). Available online at: https://selzy.com/en/blog/international-womens-day-marketing-ideas/ (Accessed June 20, 2026).

[ref22] LeSavoy B. Jordan G. (2013). The capitalist hijacking of international women’s day: Russian and American considerations. J. Int. Womens Stud. 14:244–258. Available online at: https://vc.bridgew.edu/jiws/vol14/iss3/17

[ref23] Mama A. (1995). Feminism or femocracy? State feminism and democratisation in Nigeria. Africa Dev. 20:37–58. Available online at: https://www.jstor.org/stable/43657968

[ref24] Mba N. E. (1982). Nigerian Women Mobilized: Women’s Political Activity in Southern Nigeria, 1900–1965. Berkeley: Institute of International Studies, University of California.

[ref25] McRobbie A. (2008). The Aftermath of Feminism: Gender, Culture and Social Change. London: SAGE Publications.

[ref26] Moses-Ashike H. (2022) SDG3: Access Bank’s ‘W Initiative’ to Impact 5000 Women. Available online at: https://businessday.ng/news/article/sdg3-access-bank-w-initiative-to-impact-5000-women/ (Accessed June 8, 2025).

[ref27] News Online Nigeria. (2026). Zenith Bank, Ford Foundation Honour 19 Nigerian Women with Inaugural ‘Woman of Power’ Award. Available online at: https://newsonlineng.com/zenith-bank-ford-foundation-honour-19-nigerian-womenwith-inaugural-woman-of-power-award/ (Accessed May 20, 2026).

[ref28] NewsDigestNG. (2022). IWD 2022: Gender Bills in Nigeria, The Protest and Aftermath. Available online at: https://newsdigest.ng/iwd-gender-bills-nigeria/ (Accessed December 5, 2024).

[ref101] Onwenu O., (1992). Peace Song https://www.youtube.com/watch?v=K_JY7_HntMI. Available Online at (Accessed August 17, 2025).

[ref29] Orisadare M. A. (2019). An assessment of the role of women Group in Women Political Participation, and economic development in Nigeria. Front. Sociol. 4:52. doi: 10.3389/fsoc.2019.00052, 33869375 PMC8022554

[ref30] Promasidor (2025). Promasidor Nigeria Celebrates International Women’s Day. Available online at: https://promasidor.com/en/blog/promasidor-nigeria-celebrates-international-womens-day/ (Accessed June 5, 2025).

[ref31] PunchNews. (2026). 197 Women, Girls Killed in Gender-Related Violence in 2025 — Report. Available online at: https://punchng.com/197-women-girls-killed-in-gender-related-violence-in-2025-report/ (Accessed on May 20, 2026).

[ref32] Tamale S. (2020). Decolonization and Afro-Feminism. Ottawa: Daraja Press.

[ref33] Udenigwe O. Aubel J. Abimbola S. (2026). A decolonial feminist perspective on gender equality programming in the global south. PLoS Glob. Public Health, 1–29. doi: 10.1371/journal.pgph.0005556 PMC12788186 41499422

[ref34] United Nations. (2026). International Women’s Day 8 March. Available online at: https://www.un.org/en/observances/womens-day (Accessed May 5, 2026).

[ref35] Van Allen J. (1972). ‘Sitting on a man’: colonialism and the lost political institutions of Igbo women. Can. J. Afr. Stud. 6, 165–181. doi: 10.1080/00083968.1972.10803664

[ref36] VanguardNews. (2025). Women Groups Protest Senator Natasha’s Suspension. Available online at: https://www.vanguardngr.com/2025/03/women-groups-protest-senator-natashas-suspension/ (Accessed April 20, 2026).

[ref37] World Economic Forum. (2025) Global Gender Gap Report 2025 Geneva World Economic Forum. Available online at: https://www.weforum.org/publications/global-gender-gap-report-2025/ (Accessed January 17, 2026).

[ref38] Zenith Bank. (2026). Zenith Bank International Women’s Day Seminar: Take It, You Own It. Available online at: https://digital.zenithbank.com/iwd/2026.html (Accessed March 20, 2026).

